# Predictive Equations Using Regression Analysis of Pulmonary Function for Healthy Children in Northeast China

**DOI:** 10.1371/journal.pone.0063875

**Published:** 2013-05-07

**Authors:** Ya-Nan Ma, Jing Wang, Guang-Hui Dong, Miao-Miao Liu, Da Wang, Yu-Qin Liu, Yang Zhao, Wan-Hui Ren, Yungling Leo Lee, Ya-Dong Zhao, Qin-Cheng He

**Affiliations:** 1 Department of Biostatistics and Epidemiology, School of Public Health, China Medical University, Shenyang, Liaoning Province, PR China; 2 Department of Biostatistics, School of Public Health, Saint Louis University, Saint Louis, Missouri, United States of America; 3 Department of Ambient Air Pollution Monitor, Shenyang Environmental Monitoring Center, Shenyang, Liaoning Province, PR China; 4 Institute of Epidemiology and Preventive Medicine, College of Public Health, National Taiwan University, Taipei 100, Taiwan; 5 Institute of Respiratory Diseases, the First Affiliated Hospital of China Medical University, Shenyang, Liaoning Province, PR China; Cardiff University, United Kingdom

## Abstract

**Background:**

There have been few published studies on spirometric reference values for healthy children in China. We hypothesize that there would have been changes in lung function that would not have been precisely predicted by the existing spirometric reference equations. The objective of the study was to develop more accurate predictive equations for spirometric reference values for children aged 9 to 15 years in Northeast China.

**Methodology/Principal Findings:**

Spirometric measurements were obtained from 3,922 children, including 1,974 boys and 1,948 girls, who were randomly selected from five cities of Liaoning province, Northeast China, using the ATS (American Thoracic Society) and ERS (European Respiratory Society) standards. The data was then randomly split into a training subset containing 2078 cases and a validation subset containing 1844 cases. Predictive equations used multiple linear regression techniques with three predictor variables: height, age and weight. Model goodness of fit was examined using the coefficient of determination or the R^2^ and adjusted R^2^. The predicted values were compared with those obtained from the existing spirometric reference equations. The results showed the prediction equations using linear regression analysis performed well for most spirometric parameters. Paired t-tests were used to compare the predicted values obtained from the developed and existing spirometric reference equations based on the validation subset. The t-test for males was not statistically significant (*p*>0.01). The predictive accuracy of the developed equations was higher than the existing equations and the predictive ability of the model was also validated.

**Conclusion/Significance:**

We developed prediction equations using linear regression analysis of spirometric parameters for children aged 9–15 years in Northeast China. These equations represent the first attempt at predicting lung function for Chinese children following the ATS/ERS Task Force 2005 guidelines on spirometry standardization.

## Introduction

Lung function tests have become an indispensable tool for clinical evaluation of respiratory health and diseases [1]. Spirometry is a relatively simple, non-invasive method for measuring the ﬂow and volume of air in the lung at maximal inﬂation as a function of time using forced manoeuvres [2]. It is widely accepted as a clinical tool for diagnosing obstructive, restrictive or mixed ventilatory defects such as chronic obstructive pulmonary diseases, interstitial lung diseases and asthma [3]. Spirometric reference values were obtained using predictive equations for normal healthy non-smokers. Spirometry maneuver, instruments or technologists and predictive equations may vary with study populations [4]. It is imperative to develop population-specific spirometric prediction equations to ensure the reliability of the lung function evaluation [5]. There have been several studies on spirometric reference values for healthy children in Hong Kong [6]–[7], Taiwan [8]–[9] and Singapore [10]. Pulmonary function parameters are known to vary with age, sex, height, weight, race, socioeconomic environment and geographic location [11]–[13]. The predictive equations based on these studies may not be applicable to the Han Chinese children, which accounted for 89.15% of the Chinese children [14]. Additionally, the populations used in those predictive equations were from the 1980s and were relatively small [15]. Since the mid-1990s, Chinese society has changed considerably. Growing industrialization and urbanization has led to the Chinese society more exposed to western goods and culture. Accordingly, there may be changes in lung function that may not be precisely predicted by the existing reference equations [16], and therefore there is an urgent need to develop equations that would more accurately predict spirometric reference values for Chinese children. The current study considered a nonhospital-based pediatric population used in the Northeast China Survey of Children's Health (NCSCH).

## Materials and Methods

### Ethics statement

The procedures followed were in accordance with the ethical standards of the responsible committee on human experimentation of China Medical University. All human experiments performed were approved by the China Medical University Institutional Human Ethics Committee and were consistent with the principles outlined in NIH guidelines on the ethical conduct of human research. A written informed consent was obtained from the parent/guardian of each participant before data collection.

### Study population

Samples were selected from the urban population consisting of more than 20-million residents from across 14 cities in Liaoning province in Northeast China from 2011–2012. A cross-sectional survey design was conducted in full compliance with the ethical standards of the Committee on Human Experimentation of China Medical University. These 14 cities were stratified into 3 socioeconomic zones, i.e., low, medium, and high by GDP of 2008–2010 provided by Liaoning Provincial Bureau of Statistics. In April 2009, five cities were randomly selected from these three zones, including Benxi (low zone), Anshan (medium zone), Dandong (medium zone), Shenyang (high zone) and Dalian (high zone). The numbers of the urban districts in the five cities were 3, 3, 3, 5, and 4, respectively. One elementary school and one middle school were randomly selected from each district of each selected city, resulting in a total of 36 schools each within 1.5 km of an air pollution monitor. Because we had found it difficult for many children younger than 9 years to perform the manoeuvers required for spirometry and reach the end-of-test requirements whereas most children older than 9 years often performed acceptable spirometry, we chose 9 to 15-year children from elementary and middle schools as our study sample. 150 to 200 schoolchild aged 9–15 years were then randomly selected from each of these schools, and each selected schoolchild was asked to give a questionnaire handout attached to an endorsed consent form and a return envelope to their parents or guardians to fill out. Parents or guardians of the children who wished to complete the questionnaire at home would have their children return the completed questionnaire in an envelope to the teacher. Finally, a total of 4,542 children (2,322 boys and 2,220 girls) with an average age of 12.08 years completed the survey and examination, yielding an overall response rate of 86.8%. In our study there were only 30 self-reported child smokers that accounted for a very small proportion of our study population. Finally, 3,922 healthy children (1,974 boys and 1,948 girls) were selected at random using the following GAP Conference criteria. The exclusion criteria were: past or current active tobacco smoking history, common cold within the last 4 week, history of chest injuries, and present acute diseases and past or present chronic respiratory diseases such as self-reported or medical doctor-diagnosed asthma, wheezing, persistent cough, persistent phlegm, allergic rhinitis, pulmonary tuberculosis, pneumonia, or bronchitis [17].

### Questionnaire design

Each schoolchild aged 9–15 years was asked to give a questionnaire handout attached to an endorsed consent form and a return envelope to their parents or guardians to fill out. After reviewing the questionnaire and completing the endorsed consent form, parents/guardians were invited to a Parents' Night to learn detailed information about the survey, including the objective of the survey and instructions on filling out the questionnaire. Parents who wished to complete the questionnaire at home would have their child return the completed questionnaire in an envelope to the teacher. Clinical assessment was performed via questionnaire on various respiratory problems or related diagnoses and smoking status using the American Thoracic Society Epidemiologic Standardization Project in Chinese translation [18], which had demonstrated acceptable sensitivity and specificity in school-age children in other studies conducted in China [19]–[20]. The validity of the completed questionnaire was determined by trained investigators.

Children's respiratory conditions were determined from the responses to questionnaires, categorized as follows [19]: ***a*** *)* ***Doctor-diagnosed asthma:*** defined as having been diagnosed with asthma by a doctor. ***b*** *)* ***Current wheeze:*** defined as wheezy or whistling sound from the chest, either with or without a cold. ***c*** *)* ***Persistent cough:*** defined as ever having experienced a cough on most days (≥4 days per week) for at least 3 months during the last 12 months, either with or without a cold. ***d*** *)* ***Persistent phlegm:*** defined as the mucus produced by the chest, and particularly that is expelled by coughing (sputum) on most days (≥4 days per week) for 3 months during the last 12 months, either with or without a cold. ***e*** *)* ***Allergic rhinitis:*** defined as ever having been diagnosed with allergic rhinitis by a doctor. ***f*** *)* ***Pneumonia:*** defined as ever having been diagnosed with pneumonia during the last 12 months by a doctor. ***g*** *)* ***Bronchitis:*** defined as ever having been diagnosed with bronchitis during the last 12 months by a doctor.

### Measurement

Demographic information including age, gender and smoking status (for children entering puberty) [21] was collected. Anthropometric values (weight, height, waist and hip circumference) were measured, preferably in the morning, by a nurse or physician using a standardized procedure [22], where children wore lightweight clothing and were barefoot. Each anthropometric measurement was performed twice and the average was used for the analysis. The standing height (in centimeters) of a child was measured using a Holtain's stadiometer and rounded to the nearest tenth of a centimeter. Weight (in kilograms), rounded to the nearest 100 g, was equally distributed between the two feet, head, back and both hips, and measured, using a manual SECA scale. All instruments were made in China and calibrated regularly.

Lung function values, including FEV1, FVC, MMEF, PEF and FEV_1_/FVC (%), were measured with two portable electronic type spirometers (Spirolab, MIR, Italy) by two experienced researchers and technicians. After the child was able to comprehend and follow the instructions given, spirometry was performed on the child using the ATS (American Thoracic Society) and ERS (European Respiratory Society) standards [23]. The child was asked to stand comfortably and wear a nose clip to stop air from moving through the nose during the test, and then values of total lung capacity (TLC) and a residual volume (RV) were measured on the child. Both FVC and FEV1 should be the largest value obtained from any of 3 technically satisfactory curves and the FVC and FEV1 values in at least 2 of these three curves should vary by no more than 5%. All the measurements were automatically corrected for body temperature and pressure saturated (BTPS). EpiData Entry was used for data entry and data documentation, including double entry verification, list of ID numbers in several files, codebook overview of data, and date added to backup and encryption procedures.

### Statistical analysis

Continuous variables (e.g. age) were reported as the mean ± standard deviation (SD), and categorical variables (e.g. gender) as the percentage in each subgroup. The means for age, height, weight, FVC, FEV_1_, PEF and MMEF were stratified by gender. The t-test was used to test for the differences in measurements between males and females at the level of significance of 0.01. Pearson's correlation coefficients between spirometric parameters (FVC, FEV_1_, PEF, and MMEF) and age, height and weight were calculated. The relationship between lung volumes and anthropometric variables was assessed by fitting linear, quadratic polynomial, power, and logarithmic regression models using FVC, FEV_1_, PEF and MMEF as the outcome variables and age, height, weight as the predictor variables, stratified by gender. The goodness of fit of a model was assessed by the coefficient of determination (R^2^) and residual standard deviation (RSD) [23]. Model assumptions were checked for normality, linearity, and homogeneity and independence of random errors using residual plots. Unusual and influential observations were examined, and values that caused a small, inconsequential influence on the model fit after being excluded from the model, would be retained in the model [24]. Finally, simple linear models were found to provide the best fit.

The data was randomly split into a training subset containing 2078 cases and a validation subset containing 1844 cases. The predicted values were compared with those obtained from the existing predictive equations based on the validation subset using the paired t-tests [25]. Each analysis was stratified by gender and performed by SPSS. Each test was two-tailed using a significance level of 5%.

## Results

### Demographics

The frequency and percentage for age stratified by gender was given in Table 1. The correlation coefficients between spirometric parameters (FVC, FEV_1_, PEF and MMEF) and height, weight and age were given in Table 2. All correlations were found to be positive and significant (*p*<0.001).

**Table 1:** The frequency and percentage for age stratified by gender.

Age (year)	Training set, n = 2078 Number (%)		Validation set, n = 1844 Number (%)	
	Boys	Girls	Boys	Girls
9	148 (14.1)	148 (14.4)	80 (8.6)	128 (13.9)
10	150 (14.3)	150 (14.6)	98 (10.6)	61 (6.6)
11	150 (14.3)	150 (14.6)	144 (15.6)	155 (16.9)
12	150 (14.3)	144 (14.0)	95 (10.3)	95 (10.3)
13	150 (14.3)	150 (14.6)	168 (18.1)	198 (21.6)
14	150 (14.3)	150 (14.6)	187 (20.2)	178 (19.4)
15	150 (14.3)	138 (13.4)	154 (16.6)	103 (11.2)

**Table 2:** Pearson's correlation coefficients between spirometric parameters and height, weight, age in the training subset*.

Variables	Age (year)	Height (cm)	Weight(Kg)
Boys (n = 1048)			
FVC (L)	0.745*	0.808*	0.729*
FEV1 (L)	0.776*	0.829*	0.732*
PEF (L/s)	0.709*	0.717*	0.623*
MMEF (L/s)	0.715*	0.721*	0.620*
Girls (n = 1030)			
FVC (L)	0.684*	0.769*	0.701*
FEV1 (L)	0.697*	0.767*	0.683*
PEF (L/s)	0.539*	0.590*	0.542*
MMEF (L/s)	0.596*	0.633*	0.576*

* *P*<0.001 for all correlation coefficients. FVC, forced vital capacity; FEV1, forced expiratory volume in one second; PEF, peak expiratory flow; MMEF, maximal mid-expiratory flow.

### Predictive equations based on the training set

The stepwise forward selection procedure was performed to determine the best model. Finally, the simple linear regression models with predictor variables of age, height and weight were found to provide the best fit. The estimated regression models for all spirometric parameters stratified by gender were presented in Table 3.

**Table 3:** Predictive Equations Using Regression Analysis for the Developed and other Reference Equations Based on the Training Subset.

Males (R^2^)	Females(R^2^)
FVC = −2.588+0.022height+0.013weight+0.099age (0.703)	FVC = −2.340+0.023height+0.012weight+0.055age (0.635)
FEV1 = −2.630+0.021height+0.011weight+0.108age (0.738)	FEV1 = −2.250+0.021height+0.009weight+0.064age (0.629)
PEF = −3.638+0.028height+0.017weight+0.277age (0.570)	PEF = −2.307+0.029height+0.018weight+0.101age (0.378)
MMEF = −3.185+0.022height+0.012weight+0.212age (0.577)	MMEF = −2.300+0.023height+0.014weight+0.108age (0.441)

### Predictive equations based on the validation set as compared to the existing reference equations

Table 4 compared the predicted values of FVC, FEV_1_, PEF and MMEF obtained from the developed equations and the existing reference prediction equations of Kui Feng et al. [11], Meng-Chiao Tsai et al. [8], Masato Takase et al. [26], John Hankinson et al. [27], and Mary S. M. Ip et al. [6] based on the validation subset. No significant difference was found in males, indicating the developed predictive models were comparable to the existing ones in males, whereas in females non-significance was found only for PEF.

**Table 4:** Difference Between Measured and Predicted Values Based on the Validation Subset.

	FVC*	FEV1*	PEF*	MMEF*
Males				
Measured vs. Ya-nan Ma et al.	−0.01±0.06,0.693	0.00±0.06,0.784	−0.08±0.14,0.030	0.04±0.10,0.120
Kui Feng et al.	0.69±0.07,<0.05	0.62±0.06,<0.05	1.56±0.15,<0.05	0.28±0.10,<0.05
Meng-Chiao Tsai et al.	−0.24±0.07,<0.05	−0.21±0.07,<0.05	323.98±7.73,<0.05	−0.07±0.11,0.011
Masato Takase et al.	0.50±0.06,<0.05	0.36±0.06,<0.05	0.67±0.15,<0.05	1.55±0.11,<0.05
Johnl Hankinson et al.	0.65±0.07,<0.05	0.36±0.06<0.05	0.97±0.14<0.05	−0.28±0.10,<0.05
Ip MS et al.	0.40±0.06,<0.05	0.27±0.06,<0.05		0.47±0.010,<0.05
Females				
Measured vs. Ya-nan Ma et al.	0.05±0.05,<0.05	−0.04±0.05,<0.05	−0.06±0.13,0.046	0.09±0.09,<0.05
Kui Feng et al.	0.47±0.05,<0.05	0.43±0.05,<0.05	1.82±0.13,<0.05	0.45±0.10,<0.05
Meng-Chiao Tsai et al.	−0.05±0.05,<0.05	−0.05±0.05,<0.05	286.36±5.30,<0.05	0.20±0.09,<0.05
Masato Takase et al.	0.40±0.05,<0.05	1.24± 0.05,<0.05	0.62± 0.13,<0.05	0.09± 0.09,<0.05
Johnl Hankinson et al.	0.65±0.05,<0.05	0.43±0.05,<0.05	−13.48±0.29,<0.05	14.82±0.28,<0.05
Ip MS et al.	0.38± 0.05,<0.05	0.31± 0.05,<0.05		0.70± 0.09,<0.05

* Data are presented as mean of difference (predicted-measured) ±95% CI (confidence interval) of difference, and *P* value.

## Discussion

Variables of ethnicity, gender, age, weight and height are known to have a key impact on spirometric parameters [26]. Reliable interpretation of spirometric parameters depends on the availability of predictive equations for spirometric reference values in assessing the severity and nature of functional impairments [28]. However, spirometric parameters could be overestimated or underestimated by these equations in a certain demographic population (e.g., an age-specific population) [29], and therefore it is necessary to develop predictive equations for a reference population [30]. The data used in this study measured spirometric values for an age-specific population consisting of school children aged 9–15 years in Northeast China. We developed prediction equations using multiple linear regression analysis with predictor variables of age, height, and weight. The relationship between lung volumes and anthropometric variables was assessed by linear, quadratic polynomial, power, and logarithmic models, stratified by gender. Simple linear models were finally determined to be the best predictive equations for spirometric reference values. We expect the data from this study to provide an additional value to the Global Lung Initiative in efforts to improve lung function reference values [31]–[32].

To the best of our knowledge there are only three studies in the literature which have used spirometric predictive equations for Chinese children. Ip et al. used regression equations to predict normative spirometric values for children aged 7–19 years in Hong Kong in May 1995-December, 1996 [6]. Tsai et al. used similar equations for children aged 6–11 years in Northern Taiwan in June 2004 – January 2005 [8]. Feng et al. applied the equations to an age-specific population of Korean children and adolescents in China in August-October, 2008 [11]. The limitations of those studies are that (1) they were geographic-specific and (2) used small data (with 309–852 subjects) that were unlikely to be representative and generalizable to the entire children population in China. In addition, there has been a lack of normative spirometric data for school-age Han Chinese children in recent 10 years. Since the mid-1990s, growing industrialization and urbanization has led to the Chinese society more exposed to western goods and culture. Accordingly, there may have been changes in lung function that may not be precisely predicted by the existing reference equations. The current study interviewed children born in 1997–2003 who experienced an improved standard of living by the rapid economic development in China, which has led to changes in living conditions, dietary habits, lifestyle, environment, and weight of children. In addition, the equipment and protocols used for spirometry measurement have changed and consequently, predictive equations based on the current standardization protocols for spirometry cannot accurately interpret the data. Hence, it is crucial to develop equations aimed for accurately predicting the lung functions for Chinese children. This study used the stepwise multiple regression techniques to predict spirometric parameters using predictor variables of height, weight and age, which had been positively correlated with spirometric parameters (Table 2), stratified by gender. The coefficients of determination (R^2^) in the models ranged from 0.6–0.7 for males and 0.4–0.6 for females, suggesting a fair fit, which were also comparable to those obtained from the existing reference equations (e.g., 0.67–0.75 in Tsai et al. [8], 0.59–0.81 in Connett et al. [10], 0.47–0.74 in Feng et al. [11] and 0.43–0.81 in Boskabady et al. [33]).

One study has suggested the inclusion of the poverty variable in the regression analysis of pulmonary function reduced the effect of ethnicity on pulmonary function [34]. The population in this study was obtained from five cities at different levels of socioeconomic status in Liaoning province, which was representative of Northeast China. Both the training and validation data were large enough to produce reliable results. The developed equations using regression analysis were comparable to the existing predictive equations [6], [8], [11], [26]–[27] in the sense that the paired t-test results suggested a lack of significant difference between their predicted values obtained in males (*p* value >0.01 in Table 4). The most interesting finding from the present study was that both the training and validation sets indicated the developed equations provided higher prediction accuracy than the existing equations. On the other hand, since none of the existing studies has reported type I and II errors, it is likely the spirometric values might have been overestimated or underestimated. By contrast, the current study used a random sample and the stepwise selection procedure, which was expected to provide improved validity and reliability of the results.

The current study has limitations. First, the developed predictive equations are only applicable to the age-specific population of Chinese children aged 9–15 years. Further studies are needed for Chinese children younger than 9 years and adults older than 15 years to better understand the pulmonary function in relation to demographics. Second, in China air quality in residential environment is likely to have an adverse impact on pulmonary function [35], and therefore a further study is needed to examine such effect. Third, as dictated by the nature of a cross-sectional study, we cannot address temporality between demographics and spirometric parameters, and therefore prospective studies using repeated measures of spirometric parameters over time (day, week, month or year) is an urgent need, especially during the lung growth and development [28]. Fourth, all outcomes were measured using only questionnaire responses, recall bias, where the respondent might be affected not only by the correct answer but by the respondent's memory, cannot be ruled out. Fifth, the study population was from Northeast China and was not demographically representative of the child population in China, and therefore extrapolation of the developed reference equations outside Northeast China would not be recommended. Sixth, this study only examined an urban child population and therefore the developed reference equations would not be applicable to children living in rural areas subject to different levels of air pollution than urban areas. Lastly, although there have been many predictive equations in the literature, due to the cost of comparing each of them with the developed models, only five of those equations, which had been widely referenced across North America, Japan, Taiwan and HongKong, were considered for comparison purposes.

In conclusion, our study updated normative spirometric values for the target population and developed predictive equations using linear regression analysis for Chinese children aged 9–15 years. Three striking advantages of the present study over other studies are: (1) a random sample was used, (2) a training subset of data was used to develop models and a validation subset was used to validate results, and (3) the stepwise forward selection procedure was used to determine the best model. These reference equations represent the first attempt at predicting lung function for Chinese children after the publication of the ATS/ERS task force 2005 guidelines on standardization of spirometry. The developed prediction equations can be used in both clinical practice and epidemiologic studies of comparable populations.
